# Alterations in the Rectal Sensitivity of Children With Chronic Constipation Evaluated by High-Resolution Anorectal Manometry

**DOI:** 10.7759/cureus.28835

**Published:** 2022-09-06

**Authors:** Rubén Peña-Vélez, Erick Toro-Monjaraz, David Avelar-Rodríguez, Flora Zárate-Mondragón, Jaime Ramírez-Mayans

**Affiliations:** 1 Gastroenterology and Nutrition, Instituto Nacional de Pediatría, Mexico City, MEX

**Keywords:** colon motility, neurogastroenterology, functional gastrointestinal disorders, high-resolution anorectal manometry, functional constipation

## Abstract

Introduction

Constipation is one of the most frequent chronic disorders in children and is almost always of functional etiology. Manometric alterations in anorectal sensitivity in children with chronic constipation are described in the literature; nevertheless, the impact of the duration of constipation on the parameters of anorectal manometry sensitivity is unknown.

Objective

To compare the parameters of sensitivity of high-resolution anorectal manometry (first sensation, threshold volume for urgency, and maximal tolerability) in children with chronic constipation, related to the time of evolution from the beginning of the symptoms.

Methods

This was a retrospective observational analytic study. The data of 39 children with functional constipation who were subjected to high-resolution anorectal manometry were included to evaluate constipation. The patients were divided into three groups according to the duration of constipation: <1 year; from 1 to 2 years; and >2 years. The parameters of sensitivity of the anorectal manometry were compared between the three groups and correlation tests were performed with the duration in months from the beginning of the symptoms of constipation.

Results

There was no difference between the sensitivity parameters of high-resolution anorectal manometry of the three groups; no correlation of these parameters with the time of evolution of the symptoms was found.

Conclusions

Alterations in the anorectal distensibility could develop early in the course of the disease, even from the first year of the beginning of the symptoms.

## Introduction

Functional constipation (FC) is the most frequent functional gastrointestinal disorder among the pediatric population [1], with a global combined prevalence of 9.5% (0.5% to 32%) [2], which means a common reason for a consultation with a pediatrician and pediatric gastroenterologist [3].

Children with FC present symptoms such as abdominal pain, excessive effort, hard and bulky stools, painful evacuation, and fecal incontinence, which causes significant stress for the child and family. In addition, some studies have shown that children with FC have a suboptimal life quality [4], adverse psychological effects, and somatization [5]; hence, it is considered an important problem of public health [6].

The evaluation of anorectal physiology by high-resolution anorectal manometry (HRAM) has gained importance in the evaluation of pediatric defecation disorders. HRAM gives useful and precise information about anorectal functions [7] and, hence, it is part of the diagnostic approach for children with constipation in many centers of pediatric digestive motility [8]. The most common reason for the indication of HRAM is the suspicion of Hirschsprung disease [9].

HRAM is increasingly used for the evaluation of children with FC since it provides information about basal pressure, sensibility, and defecatory dynamics [10]; however, currently, there are no data on the changes in anorectal sensibility and/or the pressure of children with FC concerning the duration of the symptoms. This study aimed at determining the differences in the parameters of anorectal sensibility according to the duration of constipation.

## Materials and methods

This was a retrospective observational analytic study. Data on 39 children from 4 to 17 years old were collected; these children were treated and analyzed by HRAM in a third-level hospital in Mexico City, Mexico (Instituto Nacional de Pediatría), between January 2017 and December 2019, with a diagnosis of FC according to the Roma IV criteria (Table 1) [11]. The children were divided into three groups according to the duration of constipation: <1 year (Group 1); from 1 to 2 years (Group 2); and >2 years (Group 3). The patients were receiving laxative treatment in the maintenance phase. All patients had received general recommendations on adequate dietary fiber intake and water consumption. No patient was taking an additional drug. Children with no reports of rectoanal inhibitory reflex or with incomplete anorectal manometry were excluded.

**Table 1:** Rome IV diagnostic criteria for functional constipation in children and adolescents

Diagnostic Criteria for Functional Constipation
Must include 2 or more of the following occurring at least once per week for a minimum of 1 month with insufficient criteria for a diagnosis of irritable bowel syndrome:
1. 2 or fewer defecations in the toilet per week in a child of a developmental age of at least 4 years
2. At least 1 episode of fecal incontinence per week
3. History of retentive posturing or excessive volitional stool retention
4. History of painful or hard bowel movements
5. Presence of a large fecal mass in the rectum
6. History of large diameter stools that can obstruct the toilet
After appropriate evaluation, the symptoms cannot be fully explained by another medical condition.

Anorectal manometry

The children received an enema of sodium lauryl sulfoacetate/sodium citrate to evacuate the rectum before HRAM. HRAM was performed on the kid on left lateral decubitus. The InSIGHT Ultima® device (Sandhill Scientific, Inc., Highlands Ranch, Colorado) and the software BioView Analysis (Abbott Molecular Inc., Des Plaines, IL) were used in this study. The parameters were evaluated and registered in the following order: pressure at rest, maximal pressure, and inhibitory rectoanal reflex. The balloon was insufflated manually to measure the parameter of sensibility (the first sensation, urge to defecate, and pain with maximal tolerability).

Statistical analysis

Descriptive statistics were used in this study. The differences between the three groups were evaluated through a one-way analysis of variance (ANOVA). A Spearman correlation proof with P<0.05 was used to correlate the parameters of HRAM and the time in months of the evolution of constipation. The statistical analyses were performed using the SPSS 22.0 software (IBM Corp, Armonk, NY).

Ethical considerations

Informed consent/assent was obtained from the parents and patients before the procedure (HRAM). The authors declare that this article does not contain personal information that allows identifying the patients. This study follows the universal ethical principles of healthcare research and was approved by the corresponding hospital committee.

## Results

Thirty-nine kids were included in this study. The average age was 9.5 ± 3.3 years old and 43.6% of the included patients were female.

Regarding the parameters of sensibility, the average volume to trigger the first rectal sensation was 57.7 ± 26.7 ml in Group 1, 93 ± 66 ml in Group 2, and 92.2 ± 58.8 ml in Group 3 (p = 0.209). Regarding the volumes to trigger the threshold volume for urgency, they were the following: 103.5 ± 35.7 ml for Group 1; 125 ± 83.9 ml for Group 2; and 137.2 ± 58.6 ml for Group 3 (p = 0.366). Lastly, to cause pain to the maximal tolerability, the volume for Group 1 was 139 ± 44.5 ml, for Group 2, it was 141 ± 91.7 ml, and for Group 3, it was 181.1 ± 56.4 ml (p = 0.154). In the same way, no differences were found in basal pressure or in maximal contraction pressure (Table 2). No correlation was found between the duration of the evolution of constipation and the values obtained from HRAM. Table 3 shows the correlation between the duration in months of constipation and the parameters of anorectal manometry.

**Table 2:** Comparison of the values of high-resolution anorectal manometry parameters based on the time of evolution of constipation

Parameters	Group 1 (n=11)	Group 2 (n=10)	Group 3 (n=18)	P-value
Resting pressure (mmHg)	44.3 ± 18.6	52.1 ± 16.5	38.8 ± 18.5	0.193
Maximum pressure (mmHg)	135.5 ± 49.5	114.5 ± 67.1	116.8 ± 92.1	0.771
First sensation (ml)	57.7 ± 26.7	93 ± 66	92.2 ± 58.8	0.209
Threshold volume for urgency (ml)	103.5 ± 35.7	125 ± 83.9	137.2 ± 58.6	0.366
Maximum tolerability (ml)	139 ± 44.5	141 ±91.7	181.1 ± 56.4	0.154
Group 1: <1 year’s duration; group 2: 1-2 years duration; and group 3: >2 years duration.				

**Table 3:** Correlation between the time of evolution (in months) from the beginning of constipation and the parameters of anorectal manometry

	Resting pressure	Maximum pressure	First sensation	Threshold volume for urgency	Maximum tolerability
r	-0.348	-0.261	0.122	0.104	0.268
p	0.055	0.156	0.513	0.579	0.146

## Discussion

In the present study, no significant differences were found in the parameters of sensitivity of HRAM, regarding the duration of time of the symptoms of FC. The hypothesis was that the alterations in anorectal distensibility could be more frequent in children with constipation with a longer duration. Nevertheless, it was found that an alteration in the anorectal distensibility, independent of the time of evolution of constipation, was registered through the parameters of sensibility of HRAM.

There are two concepts about the evolution and prognosis of constipation at the pediatric age. Some authors consider that constipation is a clinical condition that tends to remit gradually [12]. Nevertheless, it has been reported that children with FC keep experiencing symptoms in the later years and constipation could persist until adulthood [13]. A study that evaluated 418 children with FC showed that in 30% of the participants, the symptoms of constipation persisted until the age of 16 [14]. Another cohort study that included 401 children with FC reported that 25% of the patients persisted with the symptoms in adulthood; the factors associated with the persistence of the symptoms were a higher age at the beginning of the symptoms, the delay of one year of medical attention, and a lower frequency of defecation at the beginning of the treatment [15]. On the other hand, a study that observed 47 children with FC whose symptoms started in the first year of life, reported that 69% of the children that received treatment in the first three months after the beginning of the symptoms presented a normalization in the evacuation pattern, without requiring more laxatives, compared to the 32% of children that received a treatment three months or more after the beginning of the symptoms (p = 0.002) [16].

To our knowledge, this is the first study that compares the parameters of sensitivity of the HRAM in children with FC in relation to the duration of symptoms. In general, there is evidence that suggests that early intervention and a timely start with laxative medication improve the prognosis of FC in children. Our findings support this evidence since they show that there could be alterations in the anorectal distensibility (which means to support bigger rectal volumes) in the early stages of the disease, as suggested by the alterations in the anorectal distensibility observed in children from the first year of the duration of constipation. Although higher values were reported for anorectal sensitivity in Groups 2 and 3, the values of sensitivity reported for Group 1 in our study are higher than the reference values proposed by the Motility Working Group of the British Society of Pediatric Gastroenterology, Hepatology, and Nutrition [8]. Therefore, our findings support the evidence that it is important to start an adequate treatment with laxatives in an early stage of the disease.

Our research has important limitations concerning the design and characteristics of the study, which is retrospective, and the analyses with the data boil down to descriptive proof. On the other hand, a small number of patients were included, some of them with previous laxative treatment; nevertheless, the doses were not adequately established and for a time that was not greater than three months. Another limitation is that in the complementary evaluations, a neuropsychological assessment was not considered given the relationship of constipation with disorders of the gut-brain axis due to the fact that central brain activity and perception of visceral stimuli can be modulated by emotions, attention, and cognitions. However, it supports clinical observations of the findings by other studies that report that the early establishment of adequate treatment improves the long-term prognosis of constipation.

## Conclusions

In conclusion, in this study, we found that alterations in the anorectal distensibility could develop early in the course of the disease; the first changes in the anorectal sensibility could develop even during the first year of the beginning of the symptoms of constipation. Our findings support the recommendations that early pharmacological treatment has a positive impact on the resolution of constipation at the pediatric age. Children with constipation should be referred to specialized centers for care, proper evaluation, and treatment.
